# Blood brain barrier opening is not just blood-brain barrier opening

**DOI:** 10.1186/s12967-024-05431-0

**Published:** 2024-07-15

**Authors:** Deepa Sharma, Gregory J. Czarnota

**Affiliations:** Sunnybrook Research Institute and Sunnybrook Health Sciences Centre, Ontario, Canada

With great interest, we have read the recent publication of Tazhibi et al. “Focused ultrasound-mediated blood-brain barrier opening is safe and feasible with moderately hypofractionated radiotherapy for brainstem diffuse midline glioma. J Transl Med. 2024 Mar 30;22(1):320”. The authors provide intriguing data on a combination of focused ultrasound (FUS) and hypofractionated radiotherapy for the opening of the blood-brain barrier (BBB) in the preclinical diffuse midline glioma (DMG) model. They demonstrated that the opening of BBB mediated through FUS in conjunction with radiotherapy was safe and feasible in rodents bearing DMG. To date, numerous groups have extensively explored the safety and feasibility of BBB opening however, only sparse research has been conducted to understand the consequent cellular and subcellular events in brain microvascular endothelial cells. The BBB is an integral part of the human brain’s physiology, and its integrity is maintained by the microvascular endothelial cells. A deep understanding of how these cells function during BBB opening in response to ultrasound is crucial for preserving brain homeostasis.

Specifically, it is known that ceramide, a bioactive sphingolipid, and its related enzyme acid sphingomyelinase (ASMase) can act as an important second messenger in endothelial cell signal transduction. Endothelial cells are known to express 20-fold higher secretory ASMase compared to other cells, making them a potential target in radiation response [1]. Evidence has demonstrated that various stress stimuli including focused ultrasound-stimulated microbubbles (FUS+MB) or radiotherapy can alter endothelial membranes causing hydrolyzation of sphingomyelin into ceramide via ASMase, consequently causing rapid endothelial apoptosis [2]. In contrast, endothelial cells pretreated with sphingosine-1-phosphate (S1P) (ceramide antagonist) can minimize ceramide-induced cell death effects. Moreover, extensive genetic studies altering host-animal vasculature by knock-out of ASMase demonstrate inhibition of microbubble-based endothelial damage in combination with enhancement [2].

It is reported that a single treatment of FUS+MB alone or radiation alone can elevate ceramide levels in endothelial cells causing its damage and these effects are known to be synergistically higher when both these treatment modalities are combined. Recently, in CNS-based studies, Fletcher et al. studied the therapeutic response of microbubble-mediated FUS combined with radiotherapy (4 Gy) in rats bearing F98 gliomas. It was observed that the FUS combined with radiotherapy demonstrated an apoptosis increase of  93% and 396% and a vessel-associated ceramide increase of 320% and 336% as compared to FUS or radiotherapy alone, respectively [3]. Thus, the presence of ceramide is associated with a significant impairment of endothelial cell function and viability.

Recent work conducted by Chen et al. demonstrated a radiation-enhancing effect in the CNS when FUS-mediated BBB-opening was combined with radiation in animal models and demonstrated applicability in patients [4]. Mechanistically, no investigations of ceramide production were undertaken there with the underlying radiation-enhancement remaining unaddressed. Similarly, here Tazhibi et al. have only conducted hematoxylin and eosin staining with no underlying immunohistochemistry investigating mechanisms. Careful examination of Fig. 3 (normal tissue) and Figs. 4, 5 (tumour) in the combined therapy (ultrasound-exposure and radiation) panels indicates an increased frequency of cell swelling, vacuolar degeneration, and eosinophilic neurons with pyknosis - consistent with potentially increased levels of cell death. Unfortunately, no immunohistochemistry or biochemical approaches pointing to an underlying biochemical mechanism for this are present. Brainstem hemorrhage and inflammation were studied, but those are gross morphological endpoints for study, not molecular assays of endothelial cell function.

The spate of genes involved in endothelial cell exposure to ultrasound and microbubbles has been characterized including genes responsible for acid and neutral-sphingomyelinase, cell-membrane lipid biogenesis, and caspase-family members responsible for apoptotic cell death with some seeing elevations up to 202.00±27.52 (ΔΔCt±SE) after exposure in vitro (supplementary information) [5]. The work has characterized the variety of ceramide species in response to ultrasound-stimulated exposure. In addition, endothelial cell function in response to microbubble exposure has been investigated to understand the physical effects of ceramide induction by microbubbles [5]. When combining ultrasound exposure for BBB opening such effects should be considered,

In short, taken en masse emerging research is demonstrating that the ultrasound parameters used in BBB opening do more than open the BBB. Whereas grossly the BBB may recover from FUS-BBB opening morphologically, ultrasound-stimulated microbubble exposure to the vasculature and endothelial cells has further extensive biochemical and molecular effects which make its combination with radiotherapy potentially cytotoxic in terms of significant radiation enhancement.
